# Outcome of Nasal continuous positive airway pressure in neonates: A cross-sectional study

**DOI:** 10.12669/pjms.40.8.8753

**Published:** 2024-09

**Authors:** Kaneez Fatima, Sughra Zulfiqar, Ammara Farooq, Musfirah Aziz

**Affiliations:** 1 Kaneez Fatima, FCPS Neonatal Fellow Combined Military Hospital, Rawalpindi, Pakistan; 2 Sughra Zulfiqar, MRCPCH Assistant professor Paediatrics, Watim Medical College, Rawalpindi, Pakistan; 3 Ammara Farooq, FCPS Assistant Professor Paediatrics, Federal General Hospital, Federal Medical and Dental College, Islamabad, Pakistan; 4 Musfirah Aziz, FCPS Senior Registrar Paediatrics, Watim Medical College, Rawalpindi, Pakistan

**Keywords:** CPAP, Neonates, Respiratory Distress

## Abstract

**Objective:**

This study aimed to assess the efficacy of nasal continuous positive airway pressure (CPAP) in term and preterm neonates with respiratory distress by evaluating successful outcomes, identifying factors contributing to treatment failure, and documenting associated complications.

**Method:**

A comparative cross-sectional study design was employed. The research was conducted at Combined Military Hospital (CMH) Rawalpindi from November 2022 to July 2023. All consecutive neonates admitted during the specified period with respiratory distress requiring CPAP treatment and meeting inclusion criteria were enrolled. Pre- and post-CPAP respiratory distress levels, relevant biochemical markers, as well as mortality and morbidity rates were documented. Both descriptive and inferential statistical analyses were employed.

**Results:**

The mean age of the study cohort was 53.3±85.6 minutes. The average time to initiate CPAP was 82.4 ± 94.7 minutes. Mean gestational age stood at 34.68±2.8 weeks. CPAP was successful in 97% of babies. The low birth weight below 1200grams was the main factor related to failure of CPAP. The mean Downes score decreased from 5.8±1.3 before CPAP to 3.3±1.6 after 12 hours of CPAP and further to 1.85±2 after 24 hours. Significant improvements in Downes score were noted after 24 hours of CPAP usage (p < 0.05) using paired sample T-test.

**Conclusion:**

This study affirms the effectiveness of CPAP in addressing neonatal respiratory distress. The utilization of CPAP emerges as a valuable intervention that not only reduces the requirement for invasive ventilation but also exhibits the potential to alleviate morbidity and mortality rates among neonatal populations.

## INTRODUCTION

Respiratory distress (RD) is a crucial concern in newborns especially babies born prematurely. It contributes significantly to neonatal mortality, particularly in economically disadvantaged regions.^1^,^2^ Continuous Positive Airway Pressure (CPAP) has emerged as a beneficial intervention for managing respiratory distress (RD) in neonates. It is proven to be beneficial in neonates of all gestations. CPAP is a low cost, simple and non-invasive technique. It offers respiratory support by maintaining optimal lung function. CPAP administers positive pressure to the airways, within a range of 4 to 6 cm of water. This alleviates poor breathing efforts thus mitigating the risk of pulmonary complications.^1^-^3^

The endorsement by the World Health Organization (WHO) of CPAP as a treatment for preterm neonates afflicted with Respiratory Distress Syndrome (RDS) underscores its significance, particularly in developing economies.^4^ In this context, comprehensive assessment of clinical outcomes, including efficacy, risks, failure factors, and complications, is imperative to enhance the adoption of this cost-effective treatment method within resource-limited settings. The widespread application of nasal CPAP has the potential to substantially curtail neonatal mortality, particularly in resource-constrained regions.^5^,^6^

Studies report that 20 to 40 % of babies fail CPAP.^7^ Consequences of failure can be deleterious in new born babies particularly in low-income countries where CPAP might be the only available therapy for babies with RDS, as mechanical ventilation is expensive and requires expert staff.^8^ In such situation it becomes imperative to understand failure factors. This will make it possible for clinicians to make targeted interventions to improve CPAP success rates, thus improving neonatal survival. This research endeavors to evaluate the clinical outcomes of nasal CPAP among neonates with respiratory distress syndrome. The study aims to ascertain the frequency of clinical outcomes, success and failure determinants, as well as complications associated with nasal CPAP application in neonates facing respiratory distress.

## METHODS

It was a comparative cross-sectional study, conducted during nine months (Nov. 2022 to July 2023). Patients recruited through non-probability consecutive sampling technique. Informed consent was obtained.

### Ethical Approval

Institutions ethical board approved the study (IRB No. 420; dated Nov. 1^st^, 2022, CMH Rawalpindi IRB).

Sample size was calculated using WHO sample size calculator at 93.3% prevalence of respiratory distress in newborns. The calculated sample size of 97 was rounded off to 100 patients.

### Inclusion Criteria

All neonates born between gestational age of 28 weeks to 40 weeks treated with nasal CPAP for their respiratory distress & had clinical respiratory distress classified as Downes’ score > 4, saturations of below 90% on pulse oximetry, PCO_2_ <60 mm Hg on blood gas analysis, and x-ray results indicating the presence of RDS, pneumonia and transitory tachypnea among neonates (TTN).

### Exclusion Criteria

Neonates with respiratory distress secondary to congenital heart disease or other congenital anomaly like cleft lip/palate, choanal atresia and congenital diaphragmatic hernia Neonates with sepsis, cardiovascular instability and necrotizing enterocolitis.

The clinical respiratory distress score (RDS) pre and post CPAP, clinical diagnosis, pulse oximetry blood gas analysis, CXR findings, length of hospital stays along with other baseline variables recorded on a predesigned proforma. Respiratory distress pre-CPAP and post CPAP at different intervals of 4hr, 12hr and 24hr were recorded. Downes scoring system was used to assess RD. A total score of 0 suggests no distress, score of 1-4 mild distress, score of 5-7 moderate RD, and score of >7 severe distress or impending respiratory failure. Improvement in respiratory distress was considered as primary outcome while secondary outcome included length of hospital stay, duration of CPAP treatment in hours, mortality, complications related to use of CPAP (nasal damage, apnea, shock, pulmonary hemorrhage, pneumothorax and abdominal distension) and factors leading to CPAP failure.

The main variables were gestational age, birth weight, gender, mode of delivery, risk factors in pregnancy, whether two doses of antenatal steroids administered or not (if yes, time of last dose before birth), age in hours at start of CPAP therapy, temperature at admission to nursery, resuscitation required at birth. Success of CPAP was defined as; improvement of the respiratory distress as assessed by Downes score of below or equal to three, maintenance of SPO2 above 90% in room air after weaning from CPAP for about consecutive four hours and normalization of blood gases while failure was defined as need for mechanical ventilation.

### Data Analysis

Data was entered and analyzed using SPSS version-24. In descriptive statistics mean ± SD and frequency (percentage) was calculated. The success and failure rate were expressed as frequency and percentage, while pre and post CPAP respiratory distress was calculated by paired sample T-test.

## RESULTS

A total of one hundred participants were enrolled in the study. The mean age was 53.3±85.6 minutes, while the average time of CPAP initiation was 82.4±94.7 minutes. The mean gestational age was 34.68±2.8 weeks, with a corresponding mean birth weight of 2.3±0.8 kg. CPAP failure occurred in three individuals (3%), attributed to the necessity of a H2O pressure exceeding 8 cm and a FiO2 above 40%. Oligohydramnios emerged as a prominent maternal risk factor for respiratory distress, accounting for the highest proportion at 14% while urinary tract infection (UTI) prevailed as the predominant maternal infection at 8%.

**Fig.1:**
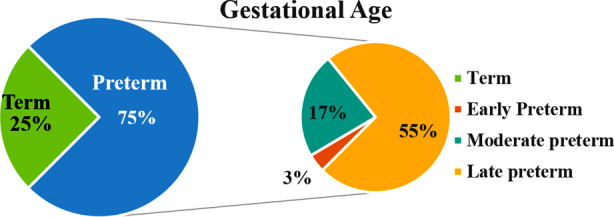
Distribution of Preterm and term neonates according to gestational age.

**Fig.2:**
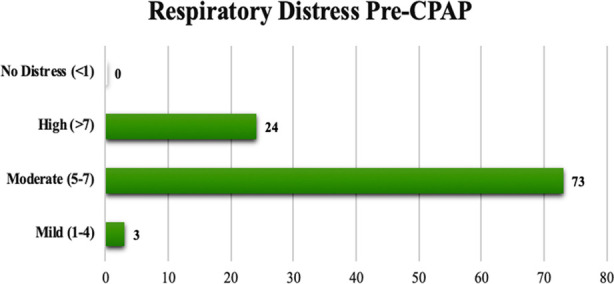
Pre-CPAP Respiratory Distress of neonates according to Downes Score.

The gestational age distribution within the study population is showcased in Fig.1. The comprehensive descriptive statistics of the study’s participant demographics are detailed in Table-I. CPAP failure was noticed in three (3%). Out of these three neonates, one (33.3%) was early preterm and two (66.7%) were moderate preterm (p=0.001). CPAP related complications were observed in 2(2.0%) of patients, both had pneumothorax, which resulted in longer hospital stay. No maternal infection and risk factor were noticed in these neonates (p=1.000). Among these neonates, the mean age at CPAP initiation was 30±25.9 minutes (p=0.333) and mean birth weight was 0.90±0.28 Kg (p=0.002). Respiratory distress pre-CPAP has shown that 73 (73%) of neonates had moderate RD, only 3(3%) had mild distress, while few neonates 24(24%) had severe distress as shown in Fig.2.

**Table-I:** Descriptive statistics of the study participants (n= 100).

Variables		Mean /n	SD / %
Age at admission (min)		53.3	85.6
Age at CPAP initiation (min)		82.4	94.7
Gestational age (weeks)		34.68	2.814
Birth weight (Kg)		2.29	0.794
APGAR Score at 1min		7.08	1.187
APGAR Score at 5min		8.40	0.876
SpO_2_		88.9895	4.20105
Duration of CPAP (days)		1.87	1.339
Length of hospital stay (days)		8.62	7.707
Mode of delivery:	Lower segment C-section (LSCS)	84	84
	Vaginal delivery (SVD)	16	16
Resuscitation required in delivery room	None	82	82.0
	Oxygen	5	5.0
	PPV*	3	3.0
Anomaly scan	Normal	95	95.0
	Abnormal	2	2.0
Maternal risk factors	GDM*	5	5.0
	PIH*	6	6.0
	Meconium-stained liquor	2	2.0
	Oligohydramnios	14	14.0
Maternal infections	Covid-19 positive	2	2.0
	UTI*	8	8.0
	PROM* >18hrs	3	3.0
Surfactant treatment given		31	31.0
Antenatal steroids given		32	32.0
Tachypnea		92	92.0
Nasal flaring		94	94.0
Grunting		91	91
Intercostal Recessions		89	89
Sternal retractions		66	66
CXR findings	TTN*	47	47.0
	MAS*	3	3.0
	RDS*	45	45.0
	Pneumonia	2	2.0
Complication	Pneumothorax	2	2.0
CPAP failure		3	3.0
Mortality		6	6

* PPV: Positive Pressure Ventilation, GDM: Gestational Diabetes, PIH: Pregnancy Induced Hypertension, UTI: Urinary Tract Infection, PROM: Prolonged Rupture of Membranes, TTN: Transient Tachypnea of Newborn, MAS: Meconium Aspiration Syndrome, RDS: Respiratory Distress Syndrome.

Paired sample T-test was used to find the association between Downes score and ABGs before and after CPAP usage in term and preterm neonates. Significant findings were observed after 24 hours of CPAP usage with Downes sore (p=0.012) and after four hours of CPAP with PCO 2 (p=0.037) as mentioned in Table-II.

**Table-II:** Association of Downes Score and ABGs level pre and post CPAP with term (n=25) and preterm (n=75) neonates (N=100).

	Respiratory distress	Gestational age	Mean ± SD	P - value
Pre-CPAP	Downes Score	Term	6.12±1.4	0.215
		Preterm	5.7±1.1	
	PH	Term	7.2±0.08	0.562
		Preterm	7.2±0.05	
	PCO_2_	Term	54.9±12.3	0.641
		Preterm	56.2±9.9	
	PO_2_	Term	47.6±12.2	0.462
		Preterm	59.6±71.5	
	HCO_3_	Term	24.2±4.0	0.776
		Preterm	24.4±3.0	
Post CPAP 4hr	Downes Score	Term	4.3±0.8	0.183
		Preterm	4.6±1.3	
	PH	Term	7.3±0.08	0.182
		Preterm	7.3±0.07	
	PCO_2_	Term	35.8±10.1	0.037
		Preterm	46.7±9.5	
	PO_2_	Term	52.6±13.8	0.801
		Preterm	55.8±27.2	
	HCO_3_	Term	21.5±3.2	0.997
		Preterm	21.5±3.9	
Post CPAP 24hr	Downes Score	Term	1.1±1.3	0.012
		Preterm	2.1±1.9	
	PH	Term	7.4±0.06	0.338
		Preterm	7.4±0.07	
	PCO_2_	Term	37.7±7.7	0.183
		Preterm	41.3±10.3	
	PO_2_	Term	50.4±13.8	0.797
		Preterm	49.0±20.7	
	HCO_3_	Term	22.39±6.0	0.201
		Preterm	24.5±5.8	

## DISCUSSION

The notable reduction in Downes score after 12 and 24 hours of CPAP administration in our study provides tangible evidence of substantial improvements in respiratory distress. Our study’s CPAP success rate of 97% aligns consistently with similar investigations carried out in diverse settings, both within Pakistan and beyond. Bano et al. reported an 84.2% success rate in mitigating respiratory distress through CPAP use.^9^ Further validation stemming from a Multan study where 83.1% of cases demonstrated favorable outcomes following early CPAP intervention.^10^

Early CPAP initiation has demonstrated both safety and efficacy in reducing the necessity for invasive mechanical ventilation and associated complications. Delay in initiating CPAP along with low uptake of antenatal steroids, scarce use of surfactant and severity of respiratory distress are identified as predictors of early failure of CPAP therapy.^11^ Majority of these babies (73%) in our study had moderate RDS and blood gases were not significantly abnormal pre-CPAP therapy. This along with early CPAP initiation at mean age of 82.4±94.7 minutes is a pivotal factor for higher success rate of CPAP therapy in our study. This aligns well with findings from Manandhar and Mathai, both of whom highlighted the effectiveness of CPAP in mitigating respiratory distress within 24 hours of application.^12^ Although only 31% babies had received antenatal steroids and 32% received surfactant therapy in our study population but this low number did not have a major impact on success of CPAP therapy. Babies weighing below 1200 gram have shown to have a higher failure rate of CPAP treatment 44.1% as compared to 37.4% in babies above 1200 grams.^13^ Same was the case in our study. The neonates who failed CPAP therapy had a mean birth weight of 900+/- 280 grams.

The birth weight and gestational age of study subjects in our study echoes with the findings in two other studies. Bano et al who reported mean birth weights of 2.32±0.37 kg, mean gestational ages of 31.24±1.99 weeks, and mean ages of 70.58±110.02 hours. Parkash et al, who documented comparable figures of mean birth weights of 2.41±2.4 kg, mean patient ages of 70.24±26.72 hours, and mean gestational ages of 36.32±2.72 weeks. These parallel findings affirm the credibility of our own observations.^9^,^14^

In another study, the CPAP failure rate was much higher (36%) than ours. 52 % of babies in this study had birth weight below 1.5 Kg and mean gestational age of 32 to 34 weeks.^15^ Very low rate of CPAP failure (3%) in our study further accentuates the potency of CPAP as a primary respiratory support strategy, effectively minimizing the requirement for more invasive interventions. The study reinforces the beneficial effects of CPAP proven already in studies done mainly in developed world. The information deduced from this study will aid the clinicians in our country to provide evidence-based care to newborn babies, though it is important to consider alternative non-invasive respiratory support modalities as well. High-flow Nasal Cannula Oxygen Therapy (HFNC) has emerged as a feasible alternative to CPAP. Although both CPAP and HFNC yield positive outcomes in alleviating respiratory distress, future studies comparing their efficacy and safety profiles have the potential to guide clinical decision-making.^16^-^18^

### Limitations

Main limitations are relatively small sample size, cross-sectional design, and single-center nature. Consequently, while the results are valuable, their generalizability is limited, and potential selection bias should be acknowledged. Advanced research methodologies, including systematic reviews and meta-analyses, are warranted to further validate these findings.

## CONCLUSION

This study demonstrates that nasal CPAP is an effective and safe treatment for respiratory distress in both term and preterm neonates and early initiation leads to a significant improvement reducing the need for invasive ventilation and associated complications. The low rate of its failure further supports its use as primary respiratory support therapy.

### Recommendations

CPAP, a low cost, simple and non-invasive technique, offers respiratory support by maintaining optimal lung function. Our findings suggest that the provision of the CPAP at every health care facility especially in neonatal centers will be beneficial for neonates presenting with respiratory distress.

### Authors Contribution:

**KF:** Conception and design of study, data collection.

**SZ:** Data analysis, interpretation of data and drafting of the article discussion. She is also responsible for accuracy and integrity of work.

**AF:** Revision of intellectual content and literature search.

**MA:** Final review.
